# Advances and prospects in biomimetic multilayered scaffolds for articular cartilage regeneration

**DOI:** 10.1093/rb/rbaa042

**Published:** 2020-09-30

**Authors:** Liwei Fu, Zhen Yang, Cangjian Gao, Hao Li, Zhiguo Yuan, Fuxin Wang, Xiang Sui, Shuyun Liu, Quanyi Guo

**Affiliations:** 1 School of Medicine, Nankai University, No. 94 Weijin Road, Nankai District, Tianjin 300071, China; 2 Beijing Key Lab of Regenerative Medicine in Orthopedics, Key Laboratory of Musculoskeletal Trauma and War Injuries PLA, Institute of Orthopedics, The First Medical Center, Chinese PLA General Hospital, No. 28 Fuxing Road, Haidian District, Beijing 100853, China; 3 Department of Bone and Joint Surgery, Renji Hospital, School of Medicine, Shanghai Jiaotong University, No. 160 Pujian Road, Pudong New District, Shanghai 200127, China

**Keywords:** biomimetic multilayered scaffold, articular cartilage, regeneration, tissue engineering, osteochondral

## Abstract

Due to the sophisticated hierarchical structure and limited reparability of articular cartilage (AC), the ideal regeneration of AC defects has been a major challenge in the field of regenerative medicine. As defects progress, they often extend from the cartilage layer to the subchondral bone and ultimately lead to osteoarthritis. Tissue engineering techniques bring new hope for AC regeneration. To meet the regenerative requirements of the heterogeneous and layered structure of native AC tissue, a substantial number of multilayered biomimetic scaffolds have been studied. Ideal multilayered scaffolds should generate zone-specific functional tissue similar to native AC tissue. This review focuses on the current status of multilayered scaffolds developed for AC defect repair, including design strategies based on the degree of defect severity and the zone-specific characteristics of AC tissue, the selection and composition of biomaterials, and techniques for design and manufacturing. The challenges and future perspectives of biomimetic multilayered scaffold strategies for AC regeneration are also discussed.

## Introduction

Articular cartilage (AC) defects, as potentially severe pathologies, have been one of the major challenges in regenerative medicine [1, 2]. There are many causes of AC defects, including trauma, ageing, disease and inflammation [3, 4]. According to the standards of the International Cartilage Repair Society, AC defects can be classified into four levels: Grade I−III defects are chondral defects, while Grade IV defects are osteochondral defects that disrupt the subchondral bone [3, 5, 6]. Due to the sophisticated hierarchical structure and limited ability of AC to self-repair, AC defects carry the risk of inducing osteoarthritis, placing a great burden on society, health care and the economy [7, 8]. 

### AC tissue anatomy

**Figure 1.**
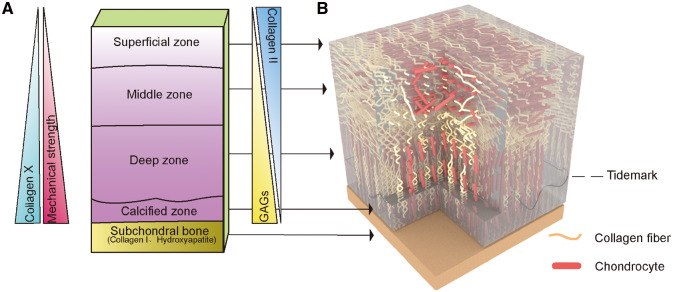
The five different layers of AC show zone-specific cell morphologies, matrix compositions, collagen fibril orientations and mechanical properties. (**A**) The content of collagen type X and GAG and compressive strain increase with depth, while the collagen type II concentration is inversely proportional to depth in the cartilaginous region. Subchondral bone is composed mainly of collagen type I and hydroxyapatite. (**B**) (1) The superficial zone has the highest density of chondrocytes and collagen fibres parallel to the joint surface. (2) The Middle zone has randomly oriented collagen fibres. (3) The deep zone has fibres perpendicular to the joint surface and tidemark, which is a basophilic line between uncalcified and calcified cartilage. (4) The calcified zone is the transition from cartilage to bone, and it has hypertrophic chondrocytes and anchors the fibres to the subchondral bone

To fully understand regeneration strategies, it is necessary to understand the anatomy and physicochemical properties of native AC tissue [9]. In fact, mature AC consists of two spatially different regions: the cartilaginous region, including the surface zone, the middle zone, the deep zone and the calcified zone; and the osseous region, including the subchondral bone zone [10, 11]. As shown in Fig. 1, these zones have different biochemical compositions, chondrocyte phenotypes and physiological characteristics [12−14].

### Current clinical therapeutic strategies

Current clinical treatment strategies mainly comprise two major categories: nonsurgical strategies, including nonpharmacological approaches and pharmacological treatments [15−18]; and surgical strategies, including arthroscopic debridement, bone marrow stimulation methods such as microfracture (MF), autologous chondrocyte implantation (ACI) and allograft or autograft cartilage implantation [19−24]. Their limitations are listed in Table 1. Although some progress has been made with these treatments, these approaches cannot achieve ideal regeneration of the original AC structure.

**Table 1.** Categories and limitations of current clinical therapeutic strategies

Therapeutic strategies	Categories	Specific therapies	Limitations
Nonsurgical strategies	Nonpharmacological approaches	Weight management; kinesiotherapy; physiotherapy; self-management and education [15, 16]	Palliative [15, 16]
	Pharmacological treatments	Nonsteroidal anti-inflammatory drugs (NSAIDS); paracetamol; Cox-2 inhibitors; carotenoids and HyA [17, 18]	Severe side-effects (such as injuries to the renal, cardiovascular and gastrointestinal systems); palliative [17, 18]
Surgical strategies	Arthroscopic techniques	Joint debridement [19]	Nonideal long-term effect and high possibility of relapse [22]
		Total joint arthroplasty [19]	An invasive end-stage treatment [19]
	Bone marrow stimulation	MF [23]	Regenerated tissue is usually fibrous cartilage [23]
	Implantation	ACI [19−21]	Long recovery time and fibrous tissue formation [20, 21]
		Autografts [19]	Limited graft availability [19]
		Allografts [19, 24]	Immunological rejection [19, 24]

### Tissue engineering and stratified scaffold strategies

Current clinical treatment strategies do not provide long-term solutions for AC regeneration, but tissue engineering techniques could bring new hope [25, 26]. The basic approach to tissue engineering involves the use of scaffolds, cells, and biochemical and biomechanical stimuli [19, 27]. In recent years, a variety of synthetic or natural materials have been investigated for use as scaffolds for AC regeneration [25, 28]. Due to the outstanding ability of scaffold-based approaches to incorporate various biochemical stimuli and the excellent initial mechanical properties of such scaffolds, these approaches are considered to have been fully developed [29, 30]. However, AC is a heterogeneous tissue composed of layers with different functional and biochemical properties [31]. Therefore, the effect of using homogenous scaffolds to repair AC is suboptimal [32]. To deliver relevant zone-specific cues, stratified scaffolds have been designed based on the multilayered structure, composition and biochemical requirements of AC tissue [33, 34]. Generally, tissue engineering scaffolds can be divided into monolayered scaffolds, bilayered scaffolds and multilayered scaffolds according to the stratified strategy [35]. Monolayered and bilayered scaffolds carry only one or two cell types in corresponding biological environments, and while they are obviously still applicable in mild AC injury, they are insufficient for more severe defects, such as Grade III−IV defects [33, 36]. Therefore, substantial research on multilayered scaffolds has been conducted to mimic the multilayered structure of AC.

This review aims to summarize the current status of multilayered scaffolds designed and developed for AC regeneration. Multilayered scaffold strategies for full-thickness cartilage defects and osteochondral defects are discussed separately. In the following sections, we investigate the selection and composition of various biomaterials and predesign strategies using finite element analysis (FEA). Subsequently, we discuss preparation technologies and then consider the challenges and future prospects of promoting AC regeneration.

## Biomimetic multilayered scaffolds in AC regeneration

**Figure 2.**
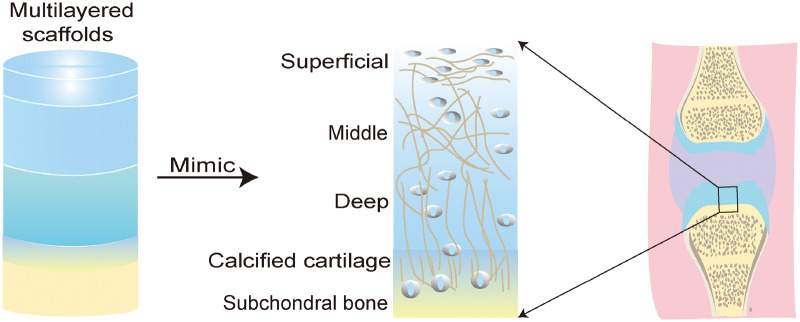
Construction of multilayered scaffolds based on the zone-specific characteristics of AC tissue

Based on the anatomical microstructure of AC and stratified scaffold strategies (Fig. 2), multilayered scaffolds can comprise three or more different compartments with dissimilar architectures made of different biomaterials [12]. Such designs achieve the purpose of mimicking the zonal structure of AC and provide new insight for the *in situ* regeneration of AC [37−39].

**Figure 3.**
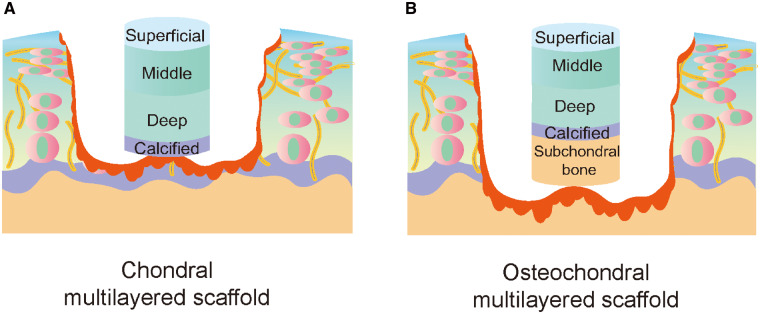
Classification of multilayered scaffolds according to the degree of defect severity. (**A**) An ideal biomimetic multilayered chondral scaffold. The surface layer is considered to protect the underlying layers from the stress in the joint, allowing cartilage repair and regeneration to occur without interference. The middle and deep layers should simulate the transition zone and the radial area of AC. The calcified layer, similar to calcified cartilage, contains biomineralization cues that make cartilage calcification and collagen type X deposition possible in this layer. (**B**) An ideal multilayered osteochondral scaffold. The construction of the cartilage layer simulates the zonal structure, and the bone layer simulates regenerated subchondral bone. The integration interface (calcified layer) between the cartilage and bone layers is very important

As shown in Fig. 3, current multilayered scaffold design strategies can be divided into two categories according to the degree of AC defect severity. The first strategy is used to repair defects involving more than two layers of cartilage or full-thickness cartilage defects; the second strategy is ideal for the repair of osteochondral defects, especially for the regeneration of subchondral bone and the integration of regenerative cartilage and subchondral bone [40−42].

### Multilayered chondral scaffolds

As shown in Table 2, chondral scaffolds need to reflect the stratified structure and zonal characteristics of the cartilaginous region, including the cell properties and phenotypes, zone-specific growth factors, matrix compositions, collagen fibre orientations and mechanical properties.

**Table 2.** Multilayered chondral scaffolds

Authors	Year	Structure			Preparation	Biomimetic contents	Study	References
		Top	Middle	Bottom				
Cell-seeded scaffolds										
Kim *et al*.	2003	PEGDA hydrogel with superficial-zone chondrocytes	PEGDA hydrogel with middle-zone chondrocytes	PEGDA hydrogel with deep-zone chondrocytes	Polymerization under UVA lamp	Cell phenotype	*In vitro*	[43]
Ren *et al*.	2016	Col II hydrogel, 2 × 10^7^ cells/ml	Col II hydrogel, 1 × 10^7^ cells/ml	Col II hydrogel, 0.5 × 10^7^ cells/ml	3DBP	Cell density	*In vitro*	[44]
Mauck *et al*.	2017	HyA hydrogel/superficial-zone chondrocytes/MSCs	HyA hydrogel/middle-zone chondrocytes/MSCs	HyA hydrogel/deep-zone chondrocytes/MSCs	Polymerization under UVA lamp	Cell phenotype	*In vitro*	[45]
Cell-free scaffolds										
Nguyen *et al*.	2011	PEG: CS: MMP-pep	PEG: CS	PEG: HA	Polymerization under UVA lamp	Matrix composition	*In vitro*	[46, 47]
Camarero-Espinosa *et al*.	2016	PLA with parallel tubular pores	PLA and sulphated CNCs	PLA and phosphated CNCs with orthogonal pores	TIPS	Matrix composition; fibril orientation	*In vitro*	[48]
Parratt *et al*.	2017	PEGDA/HAMA	PEGDA/CSMA/HAMA	PEGDA/CSMA	Layer-by-layer assembly method	Matrix composition	*In vitro*	[49]
Owida *et al*.	2018	HyA hydrogel/aligned PLA nanofibres	HyA hydrogel/random PLA nanofibres	HyA hydrogel/vertical channels	Layer-by-layer assembly method	Fibril orientation	*In vitro*	[50]
Girão *et al*.	2018	Horizontal electrospun PCL	Random electrospun PCL	Vertically aligned electrospun PCL /GO-collagen	Electrospinning; freezing	Fibril orientation	*In vitro*	[51]
Gegg *et al*.	2019	100Gel:0CS μRB (aligned+ unaligned)	90Gel:10CS unaligned μRB	75Gel:25CS unaligned μRB	Polymerization under UVA lamp	Matrix composition; fibril orientation; pore properties	*In vitro*	[52]
Munir *et al*.	2020	Aligned electrospun PCL	Random electrospun PCL	Cryo-printed helical PCL	Cryo-printing; electrospinning	Fibril orientation	*In vitro*	[53]

PEGDA, poly(ethylene glycol) diacrylate; 3DBP, 3D bioprinting; HyA, hyaluronic acid; MSCs, mesenchymal stem cells; PEG, polyethylene glycol; CS, chondroitin sulphate; MMP-pep, matrix metalloproteinase-sensitive peptides; PLA, poly(d,l-lactide); CNCs, cellulose nanocrystals; TIPS, thermally induced phase separation; HAMA, methacrylated hyaluronic acid; CSMA, methacrylated chondroitin sulphate; PCL, polycaprolactone; GO, graphene oxide; μRB, microribbon.

#### Multilayered chondral scaffolds with respect to cell properties and phenotypes

The only cell type in the cartilaginous region is chondrocytes [13, 26]. However, chondrocytes exhibit differences in properties, including cell distribution, density, size and cell phenotype, depending on the zone of cartilage tissue [12, 54]. Multilayered scaffolds can be fabricated based on the zonal properties of chondrocytes. For example, Ren *et al*. considered the zonal changes in chondrocyte density to prepare a zonal trilayered engineered cartilage construct. From the top layer to the bottom layer, the ratio of the cell densities in the three zones was 3:2:1. The results showed that simulating the zonal cell density resulted in the zonal distribution of extracellular matrix (ECM) [44].

Cell phenotype is an important and the most studied feature in cell-seeded scaffold design. In 2003, Kim *et al.* first collected cartilage slices from the upper, middle and lower regions of calf AC, isolated cells and wrapped each cell subgroup in three layers of hydrogel. After 3 weeks of culture, the histological behaviour of each layer was similar to that of native AC, which proved that chondrocytes of different layers embedded in multilayered photopolymerized gel could be used as an experimental model for zonal cartilage tissue regeneration [43]. Although the above method is feasible, the scarcity of chondrocytes is a major clinical limitation. Mauck *et al.* used the layered coculture of zonal chondrocytes and mesenchymal stem cells (MSCs) to solve this problem. They constructed a three-layered structure using the layered co-culture of zonal chondrocytes and MSCs. Moreover, they introduced porous hollow fibres to serve as channels and enable soluble factors to continuously spread to the central core. The results showed that the multilayered construct could reproduce the zonal properties of native cartilage and minimize the need for large numbers of chondrocytes [45].

#### Multilayered chondral scaffolds with respect to zone-specific growth factors

During the development of AC, zone-specific growth factors form a spatiotemporal gradient to direct MSC differentiation [27, 55]. The superficial zone is formed via the effects of transforming growth factor-β (TGF-β) and bone morphogenetic protein-7 (BMP-7) [56], while insulin-like growth factor-1 (IGF-1) plays an important role in the formation of the middle zone [57, 58]. Indian hedgehog (IHH) can promote the formation of calcified cartilage through accelerated chondrocyte hypertrophy [59]. Furthermore, from the superficial zone to the middle and calcified zones, the concentration of TGF-β1 loaded increased from 3 to 30 ng/ml [60, 61]. Moeinzadeh *et al.* encapsulated human MSCs (hMSCs) in injectable decellularized cartilage macromer hydrogel and cultured them in chondrogenic medium/TGF-β1/BMP-7 to form a superficial zone-like cellular construct. Furthermore, the hMSCs in the hydrogel were stimulated to undergo differentiation into cells with middle- and calcified-zone phenotypes in chondrogenic medium/TGF-β1/IGF-1 and chondrogenic medium/TGF-β1/IHH [62].

#### Multilayered chondral scaffolds with respect to matrix composition

The biochemical composition of AC mainly includes collagen type II and X, aggrecan and glycosaminoglycans (GAGs), including chondroitin sulphate (CS), hyaluronic acid (HyA) and keratin sulphate [63, 64]. In general, the concentration of collagen decreases from the superficial zone to the calcified zone, and the ratio of collagen type X increases gradually. In contrast, the GAG content increases with depth [65]. A biomimetic multilayered scaffold can be engineered to simulate zone-specific ECM compositions or to control the expression of ECM components. For example, Parratt *et al*. tested nine different hydrogel combinations, including poly(ethylene glycol) diacrylate (PEGDA), matrix metalloprotease-degradable peptide (MMP-pep), and methacrylated HyA and CS, to identify the optimal combinations to direct human bone marrow stromal cells to express zone-specific ECM component. Three combinations were identified, and a multilayered structure was prepared. The results showed distinct gradients of collagen expression and GAG secretion in the multilayered structure [49].

#### Multilayered chondral scaffolds with respect to fibril orientation

Concurrent with the increase in depth, collagen fibrils shift from being oriented parallel to the articular surface in the superficial zone to randomly and perpendicular to the articular surface in the middle and deep zones [66]. Owida *et al*. reported a new type of multilayered scaffold based on HyA hydrogel as the framework. The superficial layer wrapped aligned polylactic acid (PLA) nanofibres prepared by electrospinning technology, the middle layer wrapped randomly arranged fibres and the deep layer consisted of the HyA hydrogel with multiple vertical channels. The experimental results showed that the scaffold could induce the differentiation of zone-specific chondrocytes and production of zone-specific ECM [50].

#### Multilayered chondral scaffolds with respect to mechanical properties

The matrix stiffness in the superficial, deep and calcified zone of native AC tissue is 80 kPa, 2.1 and 320 MPa, respectively [67, 68]. Multilayered scaffolds can be designed to exhibit specific mechanical properties. In particular, scaffolds can regulate the proliferation and differentiation of cells to mimic zonal AC tissue through the presence of layers with different mechanical properties [69, 70]. Kenneth *et al*. made an early attempt; they constructed a stacked hydrogel system with 2% agarose at the top and 3% agarose at the bottom to culture chondrocytes. At first, the bottom layer showed greater mechanical strength. After culture for some time, the chondrocytes in the top layer expressed more collagen. Although the inhomogeneity of mechanical strength between the two layers became less obvious with the formation of matrix, the results still proved the feasibility of adjusting the mechanical strength of constructs by stratification [71].

Zhu *et al*. mixed collagen, chitosan−polycaprolactone (CH−PCL) copolymer and CS together to build a four-layered, porous scaffold that mimicked the zonal mechanical properties of the cartilage matrix. The compressive modulus and stress at 10% strain increased from the top layer to the bottom layer. At the same time, it was found that the scaffold showed a swelling index gradient and could support the seeded cells very well, suggesting that the multilayered scaffold has good potential for application in AC regeneration [72].

### Multilayered osteochondral scaffolds

As shown in Table 3, the ideal osteochondral implant should have three structural layers: a cartilage layer for the attachment, proliferation and differentiation of chondrocytes or MSCs, a bone layer with high mechanical strength, and an osteochondral interface to correspond to calcified cartilage [31, 82]. Therefore, in addition to the structural strategies related to the cartilage layer described in the last section, the biological characteristics of the interface and subchondral bone layer should also be considered [11, 34].

**Table 3.** Multilayered osteochondral scaffolds

Authors	Year	Structure			Preparation	Biomimetic contents	Study	References	
		Cartilage layer	Intermediate layer	Bone layer					
Cell-seeded scaffolds									
Schiavi *et al*.	2018	Alg/HyA hydrogel/hBM-MSCs	PLL/HyA	Alg/HAp/Hydrogel/hBM-MSCs	Spraying alternate layers	Mechanical loading	*In vitro*		[73]
Cell-free scaffolds									
Levingstone *et al*.	2016	Col I/Col II/HyA	Col I/Col II/HAp	Col I/HAp	Iterative layering	Matrix composition	*In vitro*/vivo (goat)		[74−76]
Zhang *et al*.	2017	Oriented ACECM	Compact PLGA/TCP	PLGA/TCP/collagen	LDM	Matrix composition; pore size and porosity	*In vitro*/vivo (goat)		[77]
Liu *et al*.	2018	CH/SF	CH/SF/HyA nanofibrous membrane units	CH/HAp	Iterative layering	Matrix composition; pore size and mechanical properties	*In vitro*		[78]
Jia *et al*.	2018	Oriented ACECM	Compact PLGA/TCP	PLGA/TCP	TIPS	Matrix composition; pore size; fibril orientation; mechanical properties	*In vitro*/vivo (goat)		[79]
Liu *et al*.	2019	15% GelMA	20%/3% GelMA/nHAp	30/3% GelMA/nHAp	Extrusion 3DP	Matrix composition; biodegradation rate; porosity	*In vitro*/vivo (rabbit)		[80]
Korpayev *et al*.	2020	CH/Col II	CH/Col II/nHAp	CH/Col I/nHAp	Iterative layering	Matrix composition; mechanical properties; porosity	*In vitro*		[81]

Alg, alginate; HyA, hyaluronic acid; hBM-MSCs, human bone marrow-derived mesenchymal stem cells; PLL, poly-l-lysine; HAp, hydroxyapatite; ACECM, articular cartilage extracellular matrix; PLGA, poly(lactic-co-glycolic acid); TCP, tricalcium phosphate; CH, chitosan; LDM, low-temperature deposition manufacturing; SF, silk fibroin; TIPS, thermal-induced phase separation; GelMA, gelatine methacrylate; nHAp, nano-hydroxyapatite; 3DP, 3D printing.

#### Engineering the calcified cartilage zone

The calcified cartilage zone has characteristics of both cartilage and bone and serves as the transition between the cartilage and subchondral bone [83]. For example, it contains few hypertrophic chondrocytes and collagen type X. In addition, alkaline phosphatase can be found in the calcified zone [84]. Therefore, the design of the calcified cartilage layer needs to consider the characteristics of both cartilage and bone. For example, Christakiran *et al*. prepared electrospun bilayered composite mats. The first layer consisted of 70S bioactive glass, and the second layer consisted of silk. The experiments demonstrated that the bilayered structure simulated the osteochondral interface by providing a spatially confined biomimetic microenvironment [85]. Similarly, Yang *et al*. studied the potential of an icariin (Ica)-conjugated HyA/collagen (Ica-HyA/Col) hydrogel to promote bone-cartilage interface regeneration. The experimental results showed that the Ica-HA/Col hydrogel could promote the deposition of calcium salt and the synthesis of collagen type X and thus may be an ideal scaffold for the repair of osteochondral defects [86].

#### Engineering the subchondral bone zone

Mainly composed of collagen type I and hydroxyapatite (HAp), subchondral bone is filled with vessels and nerve fibres and has a stronger compressive modulus than cartilage [12, 87]. In contrast to cartilage, bone tissue consists of various cell types, including osteoblasts, osteoclasts, osteocytes, chondrocytes, endothelial cells and MSCs [31, 88]. The subchondral bone layer can be mimicked based on these characteristics.

A previous study reported the use of a CH and silk fibroin (SF) composite to prepare the chondral layer and CH and HAp to prepare the bone layer. The middle layer was constructed using nanofibrous membrane units consisting of an electrospun CH/SF/HyA composite. The results showed that the scaffold could support the growth of chondrocytes and osteoblasts and mimic the chondral, calcified and subchondral bone layers [78]. Similarly, Jia *et al.* designed a biomimetic multilayered scaffold including a cartilage layer mimicking the ECM of AC, a porous 3D-printed poly(lactic-co-glycolic acid)/β-tricalcium phosphate (PLGA/TCP) bone layer and an intermediate PLGA/TCP compact interfacial layer. The experimental results showed that the scaffold could not only serve as a template for osteochondral tissue regeneration but also form a smooth osteochondral interface with an integrated tidemark and thus has good potential for application in the field of regenerative medicine [79].

## Biomaterials for biomimetic multilayered scaffolds

**Figure 4.**
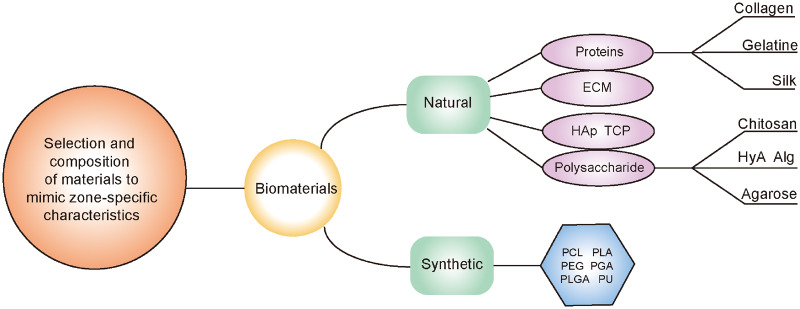
Biomaterials that are commonly applied in multilayered scaffolds

In selecting biomaterials for multilayered scaffolds, the biochemical and mechanical characteristics of the biomaterials must be fully considered in order to direct encapsulated cells to differentiate into cells present in cartilage tissue and to further induce cells to produce ECM components specific to each layer of AC [29, 89]. Strategies for selecting natural and synthetic materials based on the desired characteristics are discussed in this section (Fig. 4).

### Natural biomaterials

Natural materials used to produce biomimetic scaffolds include collagen, gelatine (Gel), CH, alginate (Alg), agarose, HyA, SF, acellular matrix and some other biological materials [90]. They have good biocompatibility and low immunogenicity, but the unstable mechanical properties and the degradation rate are also important factors affecting the use of these materials [91]. Here, we discuss how to optimize the material composition for each zone of AC.

#### Proteins

##### Collagen type I and II

Collagen is an abundant protein in mammals. It is used extensively in cartilage and bone tissue engineering [92]. Collagen type I is a structural protein that can be found in bone, and collagen type II is normally found in cartilage tissue [74, 75]. Collagen can control the phenotype of chondrocytes and osteoblasts and can be used to construct multilayered scaffolds through varying the content of different types of collagen in each layer [93, 94]. For example, Korpayev *et al*. prepared a multilayered scaffold for osteochondral regeneration made of CH/Col II (50:50 wt%, cartilage), CH/Col II/0.5% (w/v) nano-HAp (nHAp) (70:30 wt%, calcified cartilage) and CH/Col I/1% (w/v) nHAp (30 − 70 wt%, freeze-dried subchondral bone). The scaffold showed increasing mechanical strength from the cartilage to the bone layer, and coculture of the scaffold with ATDC5 and MC3T3-E1 cells resulted in the selective maintenance of chondrocyte-, hypertrophic chondrocyte- and osteoblast-specific markers [81].

##### Gelatine

Gel, the result of collagen degradation, can enhance cell surface adhesion due to the presence of bioactive motifs (l-arginine, glycine and l-aspartic acid peptides) [95]. Gegg *et al*. used CS and Gel to make macro-porous, microribbon (μRB) scaffolds, with 100Gel:0CS unaligned μRBs as the material for the superficial zone and an increasing aligned μRB content to guide cell alignment and tissue formation. Furthermore, 90Gel:10CS unaligned μRBs were used to form the middle layer, and the deep layer consisted of 75Gel:25CS unaligned μRBs. Finally, the multilayered scaffold enabled MSCs to produce cartilage with zonal biomimetic biochemical and mechanical properties after only 21 days [52].

##### Silk fibroin

SF is a natural fibrous protein that, in addition to its excellent biocompatibility, biodegradability and tuneable mechanical properties, can be applied in many fabrication methods (freeze-drying, electrospinning, 3DP, etc.) and constructed into different formats (hydrogels, films, fibres, electrospun mats, porous scaffolds, etc.) [96, 97]. These properties make it easier for SF to mix with other polymers, which facilitates the preparation of biomimetic multilayered cartilage scaffolds. For example, Zhou *et al*. produced a four-layered porous scaffold via a temperature-gradient processing method. Layers 1 − 3 were built by mixing different ratios of CH/SF composites (L1: 25:75 wt%; L2: 50:50 wt%; L1: 75:25 wt%), and the bottom layer was fabricated using a CH/nHAp composite (50:50 wt%). The *in vitro* experimental results showed that this scaffold had gradient porosity and mechanical properties similar to those of AC matrices [98].

#### Natural polysaccharides

##### Agarose

Agarose is a natural, transparent, neutral polysaccharide that is suitable for the culture of chondrocytes in terms of mechanical properties and biocompatibility [99]. Khanariant *et al*. reported an agarose hydrogel-HAp composite and tested different HAp particle sizes and doses. The results showed that 3% micro-HA in the composite was optimal for calcified cartilage formation [100].

##### Hyaluronic acid and Alg

HyA and Alg, natural anionic polysaccharides, have been widely used in cartilage regeneration [95]. Schiavi *et al*. prepared a stratified scaffold: the bottom layer was composed of Alg/HAp hydrogel, the top layer was composed of Alg/HyA hydrogel (hydrogel laden with hMSCs from bone marrow (hBM-MSCs)) and the two layers were connected by poly-l-lysine (PLL)/HyA multilayers. Furthermore, it was subjected to daily cyclic strain. The results showed that in the Alg/HA layer, the ECM was composed of GAGs and collagen type II, while collagen type X expressed by hypertrophic chondrocytes was detected in the Alg/HAp layer. Thus, this is an efficient combination of stem cells, biomaterials and mechanical loading [73].

##### Chitosan

CH, as the only natural cationic polysaccharide, can be crosslinked with many anionic polymers; at the same time, its structure is similar to that of GAG, so CH has attracted considerable interest [101]. In addition, modified CH hydrogel can provide a good chondrogenic or osteogenic environment for cells, making it an excellent choice for mimicking the zonal structures of cartilage [102]. For example, Mellati *et al*. used CH and poly(N-isopropylacrylamide) to make temperature-sensitive hydrogels with different microstrip widths for the culture of MSCs. Twenty-eight days later, the cultured cells were observed. It was found that the differentiated chondrocytes in the 50-μm-wide microstrips were similar to those in the surface region of cartilage, while chondrocytes in unpatterned constructs were similar to those in the middle region. These results showed that the hydrogel could be used for the construction of biomimetic structures [103].

#### Extracellular matrix

ECM is more attractive than other biological materials because it is tailored for cell adhesion, proliferation and differentiation in tissues and contains many related bioactive signals [104, 105]. Nasiri *et al*. prepared chemically cross-linked hybrid CH/bovine cartilage ECM scaffolds with different weight ratios by a simple freeze-drying method. It was confirmed that the presence of different ECM components improved the structure and biological characteristics of the CH scaffolds, which could be used as candidate materials for osteochondral tissue [106]. In addition, one new strategy is to structure different ECMs into multilayered scaffolds to mimic the zone-specific properties of AC tissue. For example, Cunniffe *et al*. prepared a bilayered ECM-derived scaffold [107]. The top layer consisted of AC ECM, and the bottom layer consisted of growth plate (GP) ECM. The results showed that the GP ECM layer supported the development of calcified cartilage and that the AC ECM layer supported the development of hyaline-like cartilage [108]. Overall, using layered, zone-specific ECMs to build biomimetic multilayered scaffolds is a promising approach for AC regeneration.

#### Natural bioceramics

Calcium phosphate (CaP)-based ceramics are the main natural bioceramics, and the most common forms of crystalline CaP in osteochondral tissue engineering are HAp and α-/β-TCP [109]. The structure and chemical properties of HAp are similar to those of natural apatite in bone, which allows HAp to create a microenvironment conducive to bone formation. Moreover, due to its limited mechanical properties, HAp is often used in combination with TCP [90]. These materials are used in the design of calcified cartilage and subchondral bone layers in multilayered scaffolds.

### Synthetic biomaterials

Common synthetic biomaterials that are used in osteochondral tissue engineering include PCL, polyurethane (PU), PLA, polyglycolic acid, PLGA, poly(vinyl acetate) and poly(ethylene glycol) (PEG) [95, 110]. They are easily manipulated; however, because of the wide variation in the properties of AC, composite materials or hybrid materials, such as polymer−polymer blends and copolymers, are generally used in the construction of biomimetic multilayered scaffolds [111].

#### Polycaprolactone

PCL, an FDA-approved polymer, has some drawbacks, such as a slow degradation rate and lack of bioactivity, which limit its application in tissue engineering [112]. PCL can be used in the design of biomimetic scaffolds in combination with natural polymers or other synthetic polymers [113]. For example, Zhu *et al*. grafted PCL onto the C-6 site of the CH backbone (CH-PCL) and then mixed the CH-PCL with collagen type II to prepare a four-layered scaffold. From L1 to L4, the collagen type II contents decreased from 80 to 20 wt%, whereas the PCL content in CH-PCL increased from 40 to 10 wt%. This scaffold showed a graded average pore size and porosity, swelling index and compressive modulus similar to that of AC [114].

#### Polylactic acid

PLA is another synthetic polymer that has tuneable thermal stability and degradation properties [115]. Furthermore, it has recently been tested in preparing multilayered cartilage scaffolds. For example, Camarero-Espinosa *et al*. demonstrated the fabrication of a novel multilayered polymer nanocomposite scaffold. The surface layer of this multilayered scaffold was composed of poly(d,l-lactide) (PLA) with tubular pores oriented parallel to the subchondral bone, while the middle layer consisted of PLA and sulphated cellulose nanocrystals (CNCs), and the deep layer comprised PLA and phosphated CNCs. The multilayered scaffold could emulate the corresponding features of native cartilage, including chemical cues and mechanical characteristics [48].

#### Polyurethane

PU is an elastic polymer that has good mechanical flexibility, biodegradability and tuneable chemical structures [116]. Due to these features, PU can be combined with other biomaterials to adjust the overall performance and achieve the purpose of biomimetics in tissue engineering [117, 118]. For example, Marycz *et al*. prepared synthetic biomaterials composed of PU/PLA/nHAp. After experimenting with a variety of mixing ratios, it was finally discovered that 20/80 wt% PU/PLA with 20% (w/v) nHAp was optimal for osteogenic differentiation and 80/20 wt% PU/PLA with 10% (w/v) nHAp was optimal for chondrogenic differentiation [119].

#### Poly(ethylene glycol)

PEG is widely used in cartilage tissue engineering because of its effectiveness as a scaffold or hydrogel for chondrocyte delivery [120, 121]. Moreover, PEG can be combined with various natural and synthetic materials to improve its properties. For example, Nguyen *et al*. found that the incorporation of CS and matrix metalloproteinase-sensitive peptide (MMP-pep) into PEG hydrogel (PEG:CS:MMP-pep) could induce high levels of collagen type II and low levels of proteoglycan expression, resulting in a low compressive modulus similar to that of the cartilage surface. Moreover, PEG:CS hydrogel leads to the production of intermediate levels of collagen type II and proteoglycan, similar to those in the transition zone, while PEG:HyA hydrogel induces high levels of proteoglycan production and low levels of collagen type II production, resulting in a high compressive modulus similar to that of the deep cartilage layer [46]. In a follow-up study, the researchers used the three previously studied materials to construct a three-layered biomimetic scaffold for culture with MSCs. The experimental results showed that the scaffold could effectively promote the regeneration of a multilayered, complex tissue by a single group of stem cells [47].

## Common techniques in multilayered scaffold design and manufacturing

The design and manufacture of scaffolds are two key steps in tissue engineering cartilage. According to the traditional process, the design of scaffolds requires complete *in vitro* and *in vivo* experiments to determine the optimal structural parameters and ensure the interaction of the cells with the scaffold. For multilayered scaffolds, more than two parameters need to be considered, which is a very time-consuming and labour-intensive process. Therefore, FEA has been used in regeneration of the layered tissues of AC because of its convenience in scaffold optimization [122−124].

### Design technique: FEA

FEA is a mechanical calculation tool that can divide the scaffold into small blocks with roughly regular shapes to analyse the stress at each node and predict the structural deformation, stress distribution and ability of cartilage tissue to regenerate in the composite scaffold structure, which greatly reduces the time and cost of optimizing the composite scaffold [124]. In the design of biomimetic multilayered scaffolds, it is necessary to constantly explore and improve the performance of each hierarchical structure to optimize the simulation of the biological gradient of AC, which is a time-consuming and labour-consuming process. The use of FEA provides the capability for iterative design and analysis to optimize the design of multilayered scaffolds [41].

For example, Cahill *et al*. successfully prepared porous materials with a pore size of 600 and 659 μm and a porosity of 56.4 and 55% by selective laser sintering technology. Comparison of the mechanical properties predicted by FEA showed that surface roughness and micropores have a great influence on the mechanical properties of scaffolds [125]. Koh *et al*. determined the best material properties of scaffolds for cartilage regeneration using mechanical regulation theory and a finite element model and optimized the material properties of the shallow, middle and deep areas of the scaffold model. This model played a helpful role in evaluating the scaffold design and analysing the scaffold parameters for cartilage regeneration [122].

### Manufacturing techniques

**Figure 5.**
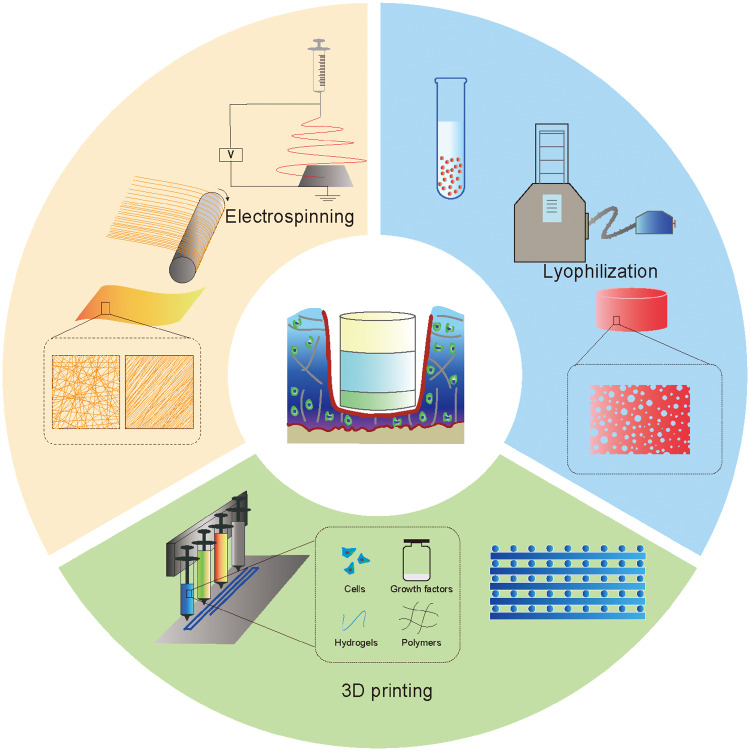
Common techniques for manufacturing multilayered scaffolds

The various properties of tissue engineering scaffolds, such as the shape, porosity and mechanical properties, are not only related to the properties of the materials used but are also closely related to the manufacturing techniques used [126−129]. Traditional scaffold manufacturing techniques, including freeze-drying, phase separation, fibre bonding and template leaching, are difficult to apply for simulating the complex multilayered microstructure of natural AC tissue [130, 131]. Therefore, certain novel techniques, or several techniques combined, are required for manufacturing multilayered scaffolds for cartilage tissue engineering (Fig. 5).

#### Multilayered lyophilization

Lyophilization, or freeze-drying, is a process that utilizes the principle of sublimation. Materials are first frozen at a very low temperature and then placed under vacuum, at which point the frozen water in the material can sublime directly from the solid phase to the gas phase, leaving a dry and porous 3D scaffold [132, 133]. As mentioned above, scaffolds manufactured using lyophilization alone cannot simulate the zonal tissue, yet lyophilizing different layers alone is also not an ideal way to construct a multilayered scaffold since it is difficult to then join these layers together. Here, we describe some multilayered lyophilization techniques.

Levingstone *et al*. prepared a biomimetic multilayered scaffold through a novel ‘iterative layering’ freeze-drying technique [74–76]. The technique consisted of repeated steps of layer addition followed by freeze-drying. The bone layer, consisting of collagen I and HAp, was fabricated through freeze-drying and crosslinking first; then 0.025 M acetic acid solution was used to hydrate the bone layer. The intermediate layer suspension, consisting of type I collagen, type II collagen and HyA, was added to the top of the bone layer and freeze-dried. Then, addition of the cartilage layer suspension, consisting of collagen type I, collagen type II and HyA, was repeated for the middle layer. The *in vivo* experimental results showed a higher level of repair in the experimental group than in the blank group, with zonal tissue regeneration, which confirms the advantages of the iterative layering technique.

Clearfield *et al*. developed a lyophilization bonding process to achieve multilayered lyophilization. First, the superficial layer was constructed by unidirectional freeze-casting a collagen type I and HyA suspension, while the lamellar osseous layer was fabricated by the coprecipitation of collagen and HyA, followed by self-compression and unidirectional freezing of the composite gel. Then, a collagen-HyA suspension, used for lyophilization bonding, as well as mimicking the transition zone of the cartilage, was placed in the middle layer to bond all of the layers together by freezing the whole construct overnight followed by lyophilization for 3 days. The multilayered scaffold was then cross-linked for further reinforcement. The results showed that the localization of HyA resembled the depth-dependent increase in GAG in native cartilage tissue. on compressive testing, the increase in stiffness with scaffold depth corresponded with that observed in normal cartilage tissue, indicating good mechanical properties [133].

#### Electrospinning

Electrospinning is a process that is used to create nanofibres from a polymer solution. Since electrospun nanofibrous structures have great potential to mimic the hierarchical architecture of ECM, this method has been extensively explored for application in tissue engineering [134−136]. More importantly, the nanofibre arrangement is tuneable. In cartilage tissue engineering, aligned nanofibres are ideal for mimicking cartilage tissue in the superficial zone with parallel fibres, while a random nanofibre arrangement resembles the collagen spread in the middle zone. Various materials can be used in electrospinning techniques, such as PCL, PLGA, PLA, SF, collagen and many other polymers [137−140]. It is appropriate to use electrospinning techniques for fabricating biomimetic multilayered scaffolds.

In one study, Munir *et al*. designed a multilayered cartilage tissue engineering scaffold consisting of three different layers: an aligned electrospun superficial zone, a random electrospun middle zone and a cryo-printed deep zone. The multilayered scaffold was found to regulate the expression of key genes compared to the controls and allowed the detection of sulphated GAG. Compared to the electrospun and control scaffolds, the multilayered scaffold also showed compressive properties more similar to those of native cartilage. Furthermore, the cryo-printed deep zone of the multilayered scaffold provided a viable initial platform for the early stage of cartilage defect repair, influencing cell attachment and load carrying [53].

Wise *et al*. constructed a directional electrospun PCL nanofibre composite scaffold for stem cell culture. The results showed that the directional electrospun nanofibres could optimize the directional ECM environment to regulate the orientation of tissue repair, and specific tissue engineering applications, such as creating the superficial area of AC, could be significantly improved by the combination of stem cells and nanofibre scaffolds [141]. Girão *et al*. proposed a method for the fabrication of 3D biomimetic, anisotropic, multilayered fibrous scaffolds. PCL was made into three kinds of bulk materials with fibres arranged horizontally, randomly and vertically through electrospinning and was then assembled with graphene oxide collagen gel to form a multilayered scaffold. The measured properties of each layer of the scaffold showed that this is a feasible method for designing and achieving fibre orientations and mechanical properties similar to those of cartilage [51].

##### 3D printing  

3D printing (3DP) is an umbrella term used to describe techniques that can be used to accurately construct prescribed 3D hierarchical structures based on computer-aided design (CAD) or computed tomography data [142]. Due to the limitations of conventional manufacturing techniques in controlling the scaffold geometry or porosity, especially for simulating zonal AC tissue, 3DP has many advantages, such as a wide range of material choices, easy processing, high porosity and a variety of achievable pore sizes. Here, we discuss some 3DP techniques frequently used in the fabrication of multilayered AC scaffolds [143−145].

Extrusion 3DP has an advantage in co-printing materials and in layered material deposition and can be used to mimic zonal AC tissue [146]. For example, Liu *et al*. constructed a biomimetic trilayered osteochondral scaffold using extrusion-based multinozzle 3DP technology; the scaffold included the cartilage layer (15% Gel methacrylate (GelMA) hydrogel), the interfacial layer (20% GelMA and 3% nHAp), and the subchondral bone layer (30%/3% GelMA/nHAp hydrogel). The construct was implanted into an osteochondral defect in the rabbit knee and showed remodelling within 3 months. Histological analysis indicated that the scaffold led to the successful repair of the rabbit osteochondral defect and thus has promise as a method for the treatment of osteochondral defects [80].

Unlike traditional 3DP techniques, low-temperature deposition manufacturing (LDM) is employed to fabricate scaffolds at temperatures below 0°C. Polymer or biomaterial solutions are deposited layer by layer in a prescribed manner based on CAD data, and then the frozen solvent is released throughout a freeze-drying process [147, 148]. Because of the low temperature, cells and growth factors are able to maintain their bioactivity during the process. More interestingly, the freeze-drying process also creates micropores in the scaffold, which are beneficial for cell attachment and proliferation [148, 149]. Zhang *et al*. developed a multilayered scaffold containing an oriented layer of cartilage matrix-CH, a compact layer of PLGA/TCP and an LDM core-sheath bone layer of PLGA/β-TCP-collagen. The whole scaffold underwent a dissolution-bonding process to assemble the three parts, and then autologous goat bone MSCs (BMSCs) were seeded into the scaffold. At 24 weeks after implantation, the femoral condyle surface was relatively flat and covered with a sufficient amount of hyaline cartilage [77]. LDM is a promising prototyping technology that is undergoing rapid development and is expected to play an important role in the production of multilayered scaffolds for cartilage tissue engineering.

3D bioprinting (3DBP) combines cell encapsulation and 3DP technologies for not only the design of biomimetic structures but also the achievement of zone-specific cell distributions by 3DP biomaterials along with cells. 3DBP can successfully achieve the layer-by-layer biofabrication process of traditional 3DP as well as print biomaterials, such as cells and growth factors, in the form of bioinks. With 3DBP techniques, the deposition of bioinks with different chemical and biological compositions can be controlled to reproduce the various zones of the AC and osteochondral interface suitably and precisely [150]. However, it is still challenging to find biomaterials suitable for use as 3DBP bioinks. Hydrogels seem to be the most promising materials for bioprinting because of their hydrophilic properties and easy incorporation of cells [151, 152].

In a recent study, Joanna *et al*. investigated a microfluidic 3DBP system. The system could deliver multiple bioinks to the extrusion head and then deposit them using a coaxial nozzle. They used the system to bioprint cell-laden hydrogel structures simulating zonal AC tissue. They demonstrated that the Alg + GelMA+ CS-methacrylated HyA (CS-AEMA) hydrogel wrapping hMSCs and human articular chondrocytes (hACs) could induce a more hyaline phenotype and that the Alg + GelMA + CS-AEMA hydrogel with methacrylated HyA (HAMA) and TCP microparticles could promote the development of hypertrophic chondrocytes [153].

## Challenges and future directions of multilayered scaffolds

According to the research mentioned above and other published work, biomimetic multilayered scaffolds are state-of-the-art strategies for stimulating the zone-specific biological and mechanical properties of native AC tissue to promote the ideal formation of both cartilage and bone layers. Advances in multilayered construct design and biomaterial selection are converging to directly enable MSCs to differentiate into a suitable phenotype in each layer and deposit zone-specific matrix [39]. It is currently impossible for a scaffold to have all the required structural features. Therefore, the successful application of biomimetic multilayered scaffolds requires sufficient signals (biological, physical and chemical) for the regeneration process to occur [37, 38, 60].

**Figure 6.**
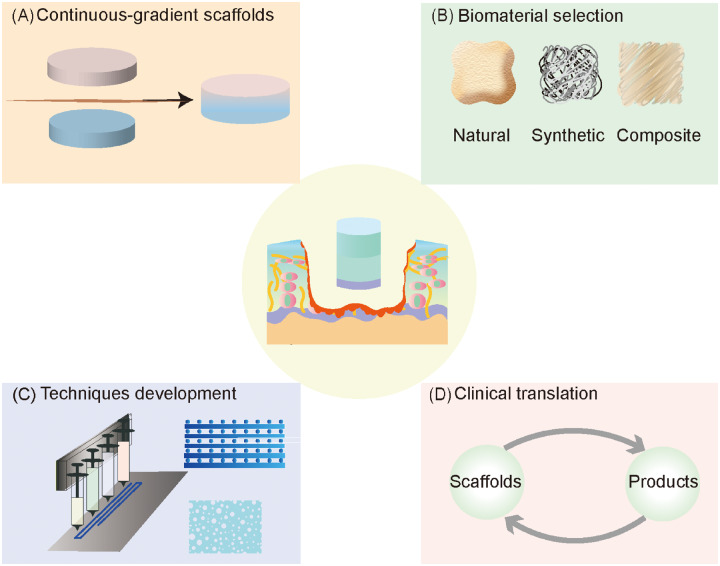
Future perspectives regarding multilayered scaffolds for AC regeneration. (**A**) The discontinuity in some multilayered scaffolds at the interface between layers limits the application of multilayered scaffolds. Continuous-gradient scaffolds will be promising with the development of fabrication technologies that can achieve a certain gradient accuracy. (**B**) Detailed knowledge of the properties of each biomaterial will help enhance the construction of biomimetic microenvironments through optimal material feature matching. (**C**) Appropriate manufacturing techniques might be beneficial in terms of the properties of multilayered constructs, such as the mechanical properties, porosity and pore size. (**D**) More importantly, future studies should explore specific and potent scaffolds with potential for successful clinical translation

One of the challenges limiting the application of multilayered scaffolds for AC repair is the discontinuity in some multilayered scaffolds at the interface between layers. At present, most multilayered constructs present discrete gradients only, while the gradients present in AC tissue are continuous [12, 31]. Especially for discontinuous stiffness gradients, the interface will be weak and susceptible to delamination under mechanical stress. Therefore, with the development of fabrication technologies that can achieve gradients with a certain accuracy, continuous-gradient scaffolds will also be promising (Fig. 6A) [12, 154]. For example, to overcome the discontinuity of mechanical properties between the layers of multilayered scaffolds, Zhu *et al*. used a gradient generation platform to fabricate a 3D PEG hydrogel with a stiffness gradient. The gradient hydrogel was used for 3D cell culture. The experimental results showed that the gradient hydrogel induced the appropriate regional behaviour of cells and promoted the deposition of zone-specific cartilage ECM [155].

The selection of appropriate composite biomaterials and the modification of their properties are also important challenges (Fig. 6B). Detailed knowledge of the properties of each biomaterial will help enhance the construction of biomimetic microenvironments through optimal material feature matching [110]. However, the limitations of specific manufacturing techniques might hamper the assembly of such environments. Therefore, the development of advanced manufacturing techniques is also crucial, as these techniques will determine the stability between the layers and the structural frame of multilayered scaffolds (Fig. 6C) [11]. Importantly, appropriate manufacturing techniques might also be beneficial in terms of the properties of multilayered constructs, such as the mechanical properties, porosity and pore size. In addition, it often takes a long time and much energy to optimize the conditions for the construction of a multilayered scaffold. The development of assistive design techniques, such as FEA, may help reduce the study cycle for multilayered scaffolds [41].

Another important question regarding multilayered scaffolds is their clinical application. Most biomimetic multilayered scaffolds are currently being tested in animals, especially small mammals (rats and rabbits), and few biomimetic scaffolds have reached the stage of clinical research [156, 157]. Specifically, Christensen *et al*. treated ten patients with osteochondral lesions with the MaioRegen^®^ scaffold. The MaioRegen^®^ scaffold is a biomimetic multilayered scaffold consisting of three layers: the bottom layer is 30% collagen type I and 70% HAp; the intermediate layer is 60/40, while the top layer consists of only collagen type I. The results showed that the treatment led to poor healing of the cartilage and subchondral bone [157]. Therefore, there is still a long way to go before successful clinical translation can be achieved (Fig. 6D).

## Conclusions

In this review, the zone-specific characteristics governing the regeneration of AC have been presented alongside an overview of multilayered scaffolds from design to manufacturing. Researchers continue to attempt to fabricate multilayered structures by understanding natural mechanisms and observing tissue development and formation. We have presented a few promising methods for the regeneration of AC tissue. In addition, multilayered scaffolds can serve as a biomimetic strategy not only for AC but also for other multilayered tissues, such as musculoskeletal cartilage. While the great promise of this strategy is acknowledged, more research is needed to achieve successful clinical translation.

## Funding

This work was supported by the National Key Research and Development Program of China (No. 2019YFA0110600) and the National Natural Science Foundation of China (No. 81772319). 

*Conflict of interest statement*. The authors declare that they have no competing interests. 
